# Distribution of Plant-Parasitic Nematodes in Michigan Corn Fields

**DOI:** 10.2478/jofnem-2023-0015

**Published:** 2023-05-02

**Authors:** Sita Thapa, Elisabeth Darling, Emilie Cole, Kristin Poley, Marisol Quintanilla

**Affiliations:** Michigan State University, Department of Entomology, East Lansing, MI 48824, United States.; Agronomist, J.R. Simplot Company, Boise, ID 83706, U.S.A; Research Manager, Corn Marketing Program of Michigan, Lansing, MI, 48906, United States.

**Keywords:** corn nematodes, detection, Michigan corn nematode identification, plant-parasitic nematode survey

## Abstract

Plant-parasitic nematodes (PPNs) can cause substantial economic yield losses to many agronomic crops in the United States. A regional-scale survey was completed across 20 counties to determine PPNs prevalence in Michigan corn and how factors such as soil type, tillage, irrigation, and cropping systems influence their distribution. Ten different major genera of PPNs were identified in Michigan corn fields: *Longidorus* (needle), *Helicotylenchus* (spiral), *Pratylenchus* (lesion), *Meloidogyne* (root-knot), *Heterodera* (cyst), *Hoplolaimus* (lance), *Tylenchorhynchus* or *Merlinius* (stunt), *Paratylenchus* (pin), *Criconemella* (ring), and *Xiphinema* (dagger). No significant differences among different categories of tillage for lesion, stunt, or needle nematode prevalence was detected. Lesion nematodes were most prevalent in muck soil, while stunt nematode prevalence was significantly affected by the soil type. Needle nematodes were least abundant in irrigated soils and in contrast, stunt nematodes were higher in non-irrigated soils. Spiral nematodes were the most common PPNs in Michigan corn in all cropping systems. These findings will be helpful in planning future nematode studies in Michigan and in developing and evaluating corn nematode management strategies.

Corn (*Zea mays* L., also called maize) is a widely cultivated crop around the world. In the United States (U.S.) economy, corn production plays a significant role in food, fuel, and fiber. With approximately 96 million acres reserved for corn production, the U.S. is the largest corn producer globally (Crop Production by State, 2021). Corn exports totaled approximately $12.9 billion in 2018 (Crop Production by State, 2021). Michigan is located at the northeast extremity of the corn belt and is the 11th largest producer of corn, with 306.5 million bushels of corn grain worth $1 billion being produced in 2020 (CropProphet, 2020; USDA/NASS, 2020). Production within the state is concentrated in the lower peninsula, with Saginaw and Lenawee counties contributing most to the state’s production values (MDARD, 2018). In Michigan, commercial corn (feed and processing) is the largest single-acreage irrigated crop (Mackellar and Kelly, 2021).

Several pests significantly impact corn production, resulting in approximately 30% yield loss globally (Oerke, 2006). Despite contributing to this production decline, nematodes have not historically received significant attention. This is in part because nematode damage is easily misinterpreted as a symptom of nutrient deficiency due to a lack of specific above-ground symptoms. Above-ground symptoms of nematode damage are stunting and/or chlorosis, while below-ground symptoms often include swollen roots, a lack of finer roots, root branching, and necrotic lesions (black or dark brown dead spots) (Tylka, 2007).

There are approximately 60 plant-parasitic nematodes (PPNs) species associated with corn in North America (Norton, 1983); however, there are just 10-12 species that are typically cited as damaging for Michigan corn (Warner, 2008). According to Koenning et al. (1999), the most common PPN genera in the U.S. are *Hoplolaimus* (lance), *Meloidogyne* (root-knot), *Pratylenchus* (lesion), *Belonolaimus* (sting), *Longidorus* (needle), and *Paratrichodorus* (stubby-root), which together can cause 5-20% yield damage in corn. In Michigan, lesion nematodes are most frequently detected (Warner, 2011); however, the corn needle nematode is the most destructive. Even a low number of needle nematodes in cornfields can cause significant damage, while many other nematodes present in corn fields cause minimal damage even at high populations (Tylka et al., 2011; Todd et al., 2016). Consequently, the needle nematode has a damage threshold of 1 individual per 100 cm^3^ of soil in corn fields (Niblack, 2003). Plant-parasitic nematodes are most often detected in plant roots and within the surrounding soil; however, needle nematodes are relatively unique because they migrate down through the soil profile as soil temperatures rise. Typically, needle nematodes are confined to fields with coarse-textured sandy soils where continuous corn or other grasses are grown (Warner, 2011).

Nematode species distribution and population density can vary among different fields. These differences can be attributed to field conditions such as soil physical and chemical properties and other environmental factors (Brodie, 1976; Norton, 1983). Additionally, management practices like crop rotation, residue management, and tillage can contribute to the abundance and diversity of both parasitic and beneficial nematode populations (Govaerts et al., 2006). In a continuous corn rotation, nematode populations may increase substantially over seasons and result in significant yield losses (Grabau and Chen, 2016). However, the effects of crop rotation on nematode population densities seem to vary with nematode genera and tillage (Govaerts et al., 2006).

Nematode distribution surveys in an economically important crop like corn are necessary to gain knowledge on risk and inform management decisions. The current distribution and risk of damage to corn due to PPNs in Michigan is poorly understood. Previous surveys in other Midwest corn-producing states, including Iowa, Ohio, and Illinois, indicated that the corn needle nematode was not the most abundant nematode species present, but instead that *Helicotylenchus* (spiral), *Pratylenchus* (pin), and *Pratylenchus* (lesion) were most prevalent (Tylka, 2011; Simon, 2018; Han et al., 2021). Although these states have similar practices, understanding the regional distribution of PPNs in Michigan cornfields and how management and abiotic factors influence these populations will provide insight into developing management tactics moving forward. Our two main objectives were: 1) to assess the abundance and distribution of important plant-parasitic nematodes in Michigan field corn, and 2) to determine the influence of crop management factors such as tillage, cropping system, irrigation, and soil type on nematode populations.

## Materials and Methods

**Figure 1:**
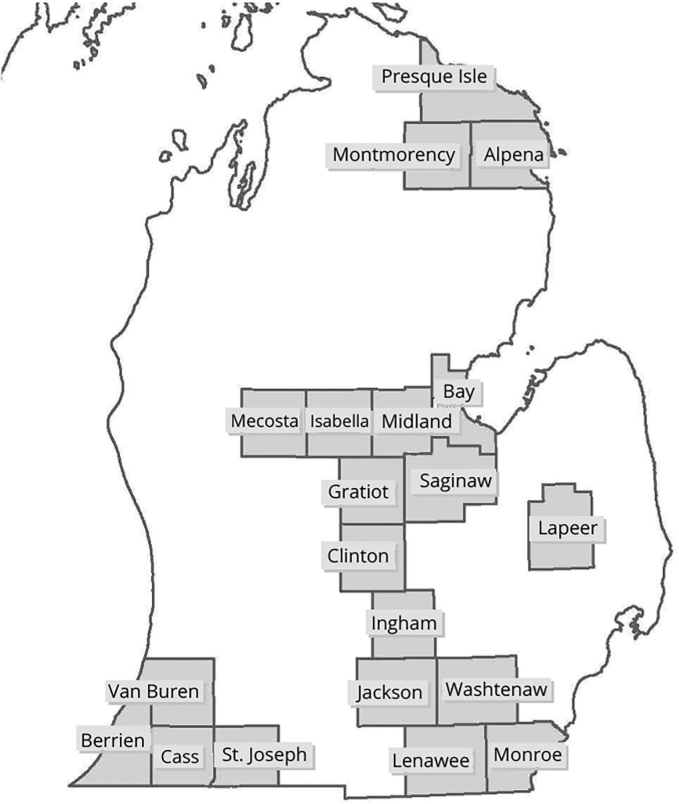
Michigan state map showing 20 counties from which soil samples were taken for 2018 study of corn nematodes.

*Soil sampling:* A regional-scale survey of plant-parasitic nematodes in Michigan corn was completed in 2018. The lower peninsula of Michigan was divided into five regions: Northwest, North, Southwest, Southeast, and Central. Corn was planted in 47 counties in Michigan (USDA NASS, 2018) of which we surveyed 20 counties, whose regional distribution was as follows: Northwest (5), North (3), Southwest (5), Southeast (3), and East/Central (4) (Fig. 1). We selected corn fields (N=86) from these regions and collected five replicate soil samples from each field twice during the season, once during May-June (V3-V5) and once post-harvest September-October. Ten soil cores were collected in a zig-zag pattern using a 2.5-cm diameter soil probe at 30-50 cm depth (Zeims, 2015). This zig-zag sampling was repeated five times within each field, twice during the season. After sampling, soil samples were transported to the Applied Nematology laboratory at Michigan State University and stored at 4°C until processed. GPS coordinates of each sampling site were recorded to map the distribution of corn nematodes. Field history data from each site was collected, including crop rotation practices, tillage practices, soil type, and nematode management strategies (if any), via communication with the grower at time of sampling. These factors were noted to detect any potential associations between nematode abundance and management practices. Soil type was collected using GPS coordinates and the USDA Web Soil Survey (USDA-NRCS, 2022). Tillage type was condensed into five categories: conventional (chisel-plowed tillage conducted every or every other season), conservative (minimal strip- or chisel-plowed every 2-3 years), no-till with exceptions, short-term no-till (<5 years of no-till), and long-term no-till (5+ years of no-till). Fields were considered no-till with exceptions if growers typically followed no-till, but tillage was conducted for one season. If tillage history was unknown, the sample was omitted for comparison.

*Nematode extraction and identification:* Each bag of soil with the ten composite soil cores was homogenized. Once homogenized, a 100 cm^3^ subsample of the sample was processed using the modified centrifugation-flotation technique (Jenkins, 1964; Ingham, 1994). Briefly, sample processing was completed by mixing with water and then pouring over stacked 250-µm and 25-µm sieves collecting nematodes on the bottom 25-µm sieve. The process was repeated three times, after which nematodes collected on the bottom 25-µm sieve were centrifuged with a 40% sucrose solution. After centrifugation, each sample was poured back into the 25-µm sieve, rinsed to remove sucrose from the nematodes, and then collected in glass test tubes. All PPNs were identified to the genus level using morphological characteristics observed with an inverted Nikon TMS microscope between 40x and 200x magnification. The morphological characteristics used for the identification were overall body shape and size, stylet shape and size, esophageal morphology, tail shape, metacorpus size, vulva position, stylet knob shape and size, nematode shape at rest position, cephalic characteristics, and other features of gross body morphology (Mai and Mullin, 1996). Plant-parasitic nematodes of each genus were counted in a gridded counting disc to determine the population density of each genus. All other nematodes (bacterivores, fungivores, omnivores, and predators) were identified as non-plant-parasitic nematodes using several keys (Hunt, 1993; Mai and Mullin, 1996). Each field included in the survey had five replicate soil samples for both dates.

*Statistical analysis:* For both sampling periods, abundance and prevalence values were determined for each nematode genus recovered in the soil survey. To calculate abundance values, each of the 86 fields with five replicates was included to determine the average count per 100 cm^3^ soil sample (N=430). Detection of ≥1 nematode of that genus recovered within any of the five field replicates was considered infested. The number of infected fields was divided by the total number of fields and multiplied by 100 to gain a percentage.

**Figure 2:**
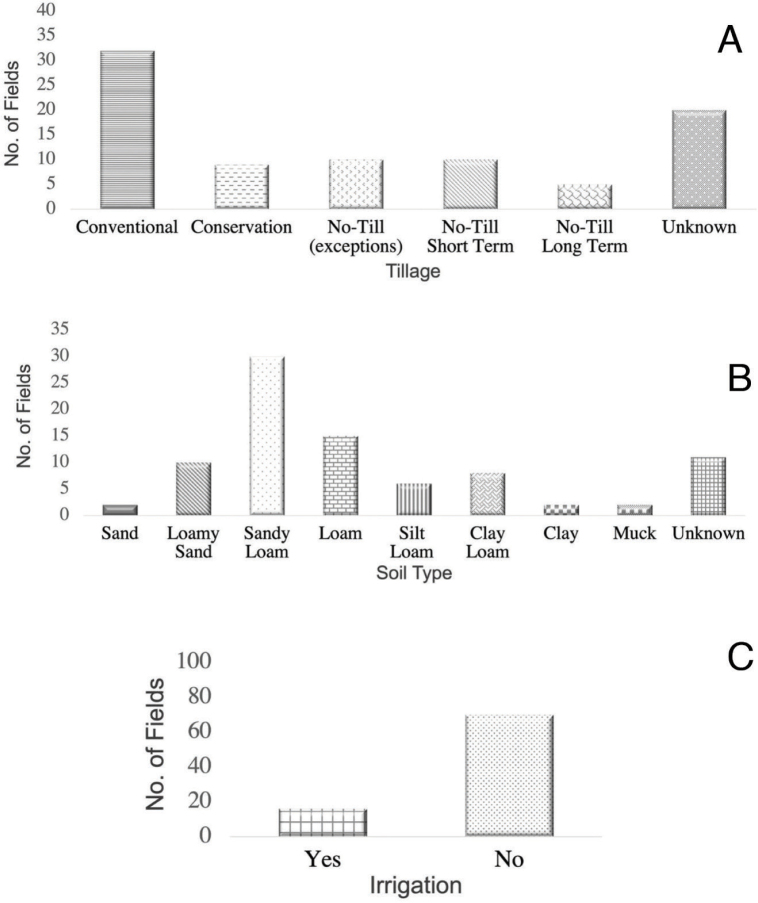
Total number of corn fields surveyed within each main category: Tillage (A), Soil type (B), and Irrigation (C).

**Figure 3:**
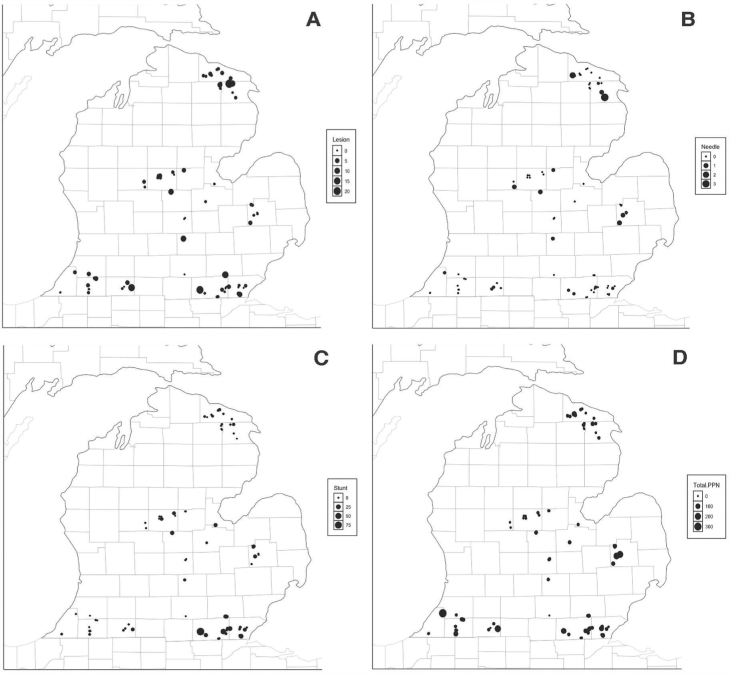
Michigan maps showing county distribution of lesion nematode (A), needle nematode (B), stunt nematode (C), and total plant parasitic nematodes (D) in corn fields in 2018. The size of the dots correlates with the number of nematodes.

For data analysis, soil sample replicates from each field at harvest were grouped together to obtain average counts per genus per field. These averages were best-fit based on their distribution. For each cropping factor, densities for each genus were analyzed. First, a Shapiro-Wilk test was used to determine if data was normally distributed and was log-transformed to limit any heterogeneity of variance (log X + 1). Generalized linear models were conducted to determine significance of soil type, tillage, crop history, and irrigation on nematode genera abundance independently. Next, we performed a Tukey HSD post-hoc analysis (α = 0.05) on lesion nematodes, stunt nematodes, needle nematodes, and total plant-parasitic nematodes (RStudio version 1.3.1093). We selected these nematode genera based on their relative abundance and their impact on corn growth. A graph showing the number of fields collected within each factor can be found in Figure 2. For the construction of needle, lesion, and stunt nematode distribution maps (Fig. 3), R packages ‘dplyr’, ‘ggpubr’, and ‘maps’ were used to visualize statewide pressure from initial soil sampling (Wickham et al., 2020; Kassambara, 2020; Becker and Wilks, 2021). Values used in these maps were obtained by averaging densities from the replicate samples (N=5) from each field, then matching that corresponding average to the GPS coordinates of each field site.

## Results

**Figure 4:**
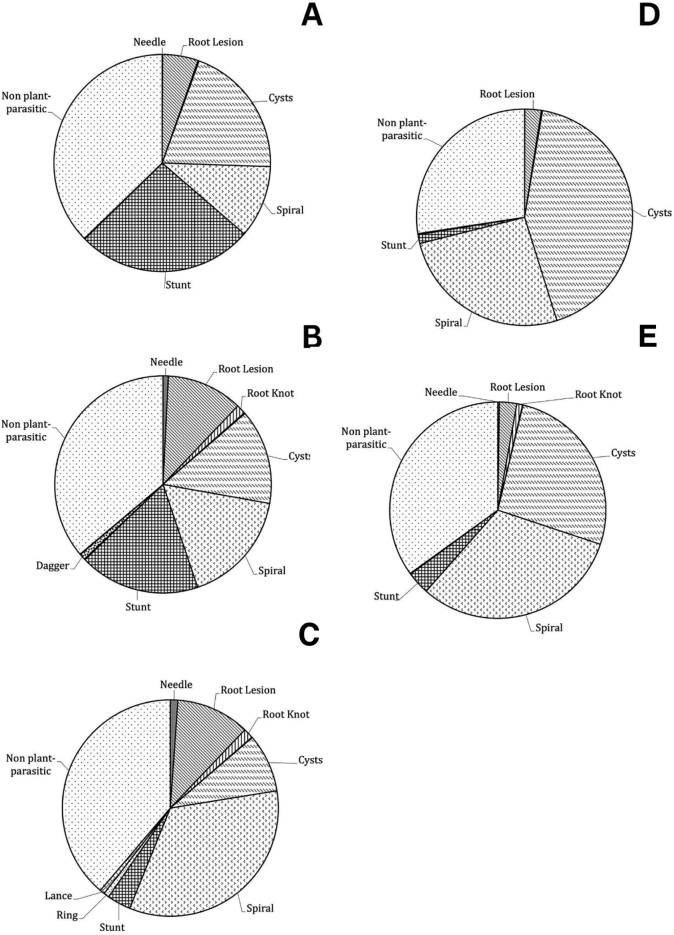
Michigan divided into five different regions for corn nematode sampling. Pie-charts show regional nematode distribution during corn harvest time in 2018. Regions: Southeast (A), Northwest (B), North (C), Southwest (D), East/Central (E).

*Plant-parasitic nematode distribution in Michigan corn regions:* Soil samples taken during harvest were analyzed for nematode distribution in harvest samples. Within these samples, we recovered ten different genera of PPNs in Michigan corn fields: *Longidorus* (needle), *Helicotylenchus* (spiral), *Pratylenchus* (lesion), *Meloidogyne* (root-knot), *Heterodera* (cyst), *Hoplolaimus* (lance), *Tylenchorhynchus or Merlinius* (stunt), *Paratylenchus* (pin), *Criconemella* (ring), and *Xiphinema* (dagger). Average individual counts per field and percentage of fields recovered are separated by nematode genus in Table 1 (Initial) and Table 2 (Harvest). Distribution maps of spiral, stunt, lesion, and needle nematodes are visualized in Figure 3 (Figs. 3A-D). These nematode genera were selected to visualize in distribution maps based on their relevance and significance in corn systems. Of the observed genera, spiral nematodes were the most prevalent and abundant nematodes within both initial and harvest samples, having been recovered from 86% and 91% of Michigan corn fields respectively (Tables 1,2). However, within the final sampling, only four corn fields contained spiral nematode densities that surpassed the threshold of 500 individuals (MacGuidin, 2003; Tylka, 2009). Regionally, spiral nematodes were most abundant in the East/Central (Fig. 4E) and North regions (Figs. 4B,C), while both stunt and spiral nematodes were present in higher numbers in the Northwest region (Fig. 3B). Lesion nematodes and stunt nematodes were the next most prevalent nematodes, having been found in 80.90% and 78.65% of cornfields respectively. Southeast Michigan corn fields contained significantly higher stunt nematode populations than the North (*P* = 0.000), Northwest (*P* = 0.001), East/Central (*P* = 0.000), and Southwest regions (*P* = 0.002). Overall, the needle nematodes were prevalent in harvest samples in 15.73% of Michigan corn fields (Table 2). On average, needle nematode distribution was impacted by region, though this was not statistically significant (*P* > 0.05). The maximum number of needle nematodes encountered was 11 per 100 cm^3^ of soil in a field located in St. Joseph County (Southwest region).

**Table 1.** Plant-parasitic nematode genus prevalence during initial sampling collected May-June 2018 in Michigan. Percentage of infested fields for each genus indicates the number of fields that contained at least one individual per 100 cm^3^ soil sample.

Genus (Common Name)	Average ± STD	Percent of Fields Infested (N=86)
*Helicotylenchus* (Spiral)	12.21 ± 31.40	86.05%
*Heterodera* (Cyst)	2.14 ± 5.60	79.07%
*Pratylenchus* (Lesion)	2.57 ± 6.53	76.74%
*Tylenchorhynchus* or *Merlinius* (Stunt)	5.12 ± 15.79	67.44%
*Longidorus* (Needle)	0.16 ± 0.65	26.74%
*Meloidogyne* (Root Knot)	0.26 ± 1.63	20.93%
*Hoplolaimus* (Lance)	0.07 ± 0.54	13.95%
*Criconemella* (Ring)	0.09 ± 0.71	10.47%
*Xiphinema* (Dagger)	0.04 ± 0.45	9.30%
*Paratylenchus* (Pin)	0.06 ± 0.59	8.13%

**Table 2.** Plant-parasitic nematode genus prevalence during corn harvest sampling collected between September-October 2018 in Michigan. Percentage of infested fields for each genus indicates the number of fields that contained at least one individual per 100 cm^3^ soil sample.

Genus (Common Name)	Average ± STD	Percent of Fields Infested (N=86)
*Helicotylenchus* (Spiral)	56.07 ± 161.50	91.01%
*Pratylenchus* (Lesion)	3.80 ± 6.08	80.90%
*Tylenchorhynchus* or *Merlinius* (Stunt)	11.00 ± 28.52	78.65%
*Heterodera* (Cyst)	10.08 ± 161.50	57.30%
*Paratylenchus* (Pin)	0.19 ± 1.65	21.35%
*Longidorus* (Needle)	0.13 ± 0.64	15.73%
*Meloidogyne* (Root Knot)	0.06 ± 0.19	14.60%
*Hoplolaimus* (Lance)	0.27 ± 1.90	10.11%
*Criconemella* (Ring)	0.12 ± 0.74	8.99%
*Xiphinema* (Dagger)	0.02 ± 0.16	3.37%

**Figure 5:**
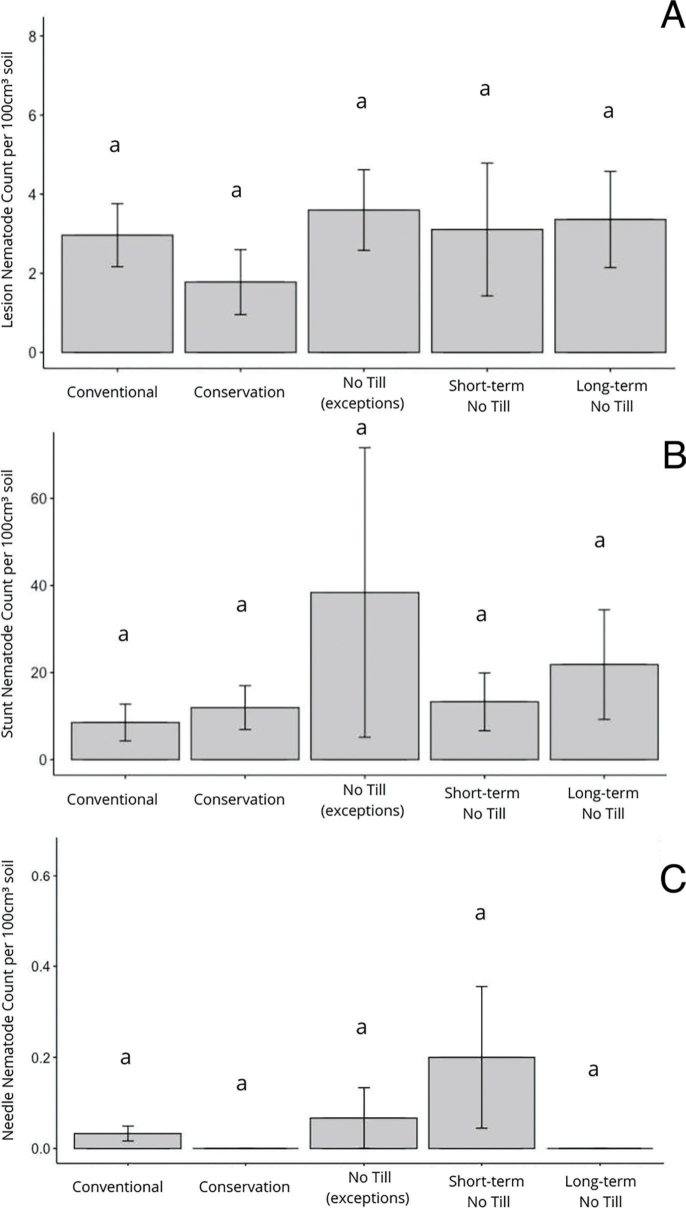
Mean population density (nematodes per 100 cm^3^ of soil) of lesion nematode (A), stunt nematode (B), and needle nematode (C) in different tillage practices in Michigan corn end-of-season samples in 2018. No significant differences were observed between nematode population densities and different tillage practices (Tukey HSD, α = 0.05).

*Plant-parasitic nematode abundance by tillage system in Michigan corn:* The primary tillage practices applied to Michigan corn fields can be categorized into conventional, conservative, no-till with exceptions, short-term no-till (<5 years of no-till), and long-term no-till (5+ years of no-till). In terms of nematode presence, there were no significant differences between any of the five categories of tillage for lesion nematode, stunt nematode, and needle nematode prevalence (Figs. 5A,C). The lowest number of lesion nematodes was found in the conservation tillage, and the highest was found in no-till (Fig. 5A). However, stunt nematode populations were highest in no-till and lowest in conventional (Fig. 5B). Similarly, short-term no-till systems had more needle nematodes than the other four types of tillage practiced in Michigan corn (Fig. 5C).

**Figure 6:**
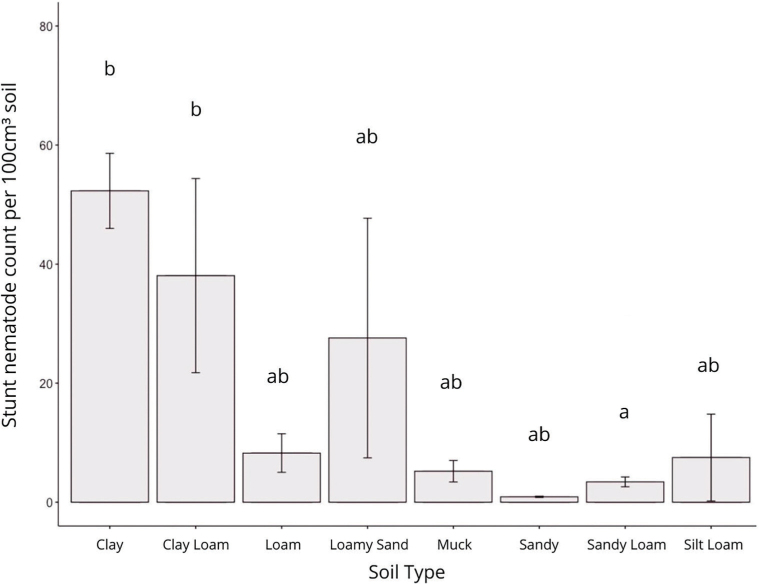
Mean population density of stunt nematodes per 100 cm^3^ of soil in different types of soil in Michigan corn grown in 2018. Letters on the top of each bar represent significant differences among the soil types (Tukey HSD, α = 0.05).

*Plant-parasitic nematode abundance by soil type in Michigan corn:* In Michigan, there are hundreds of official soil types that can be simplified into eight main categories: clay, clay loam, loam, loamy sand, muck, sandy, sandy loam, and silt loam. Among the surveyed growers, most of the growers have a loam type of soil: 42% of corn fields have sandy loam soil, followed by 17% with loam, and 13% with sandy loam. Lesion nematodes were found in all soil types; however, their populations were highest in muck soils. Alternatively, spiral nematodes were most abundant in loam soil and least in clay and sandy soils. Clay soils contained higher numbers of stunt nematodes than sandy loam (*P* = 0.04) and silty loam (*P* = 0.052) soils (Fig. 6). The highest number of non-plant-parasitic nematodes were found in clay soil, followed by clay loam; however, soil type overall did not significantly impact beneficial nematode populations (*P* > 0.100).

**Figure 7:**
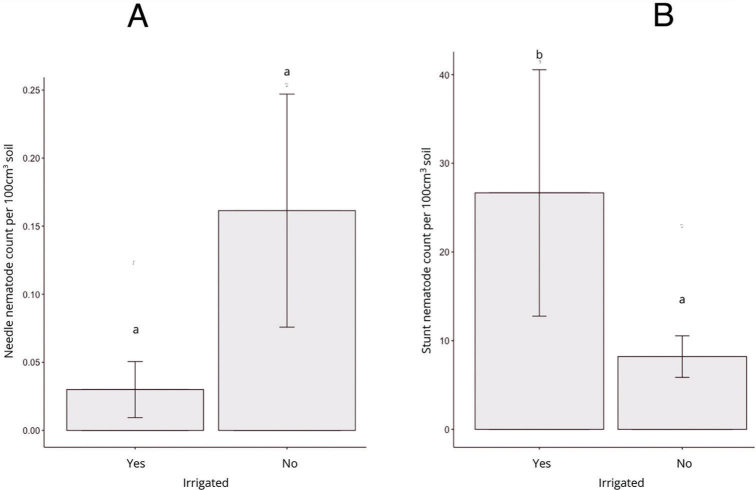
Mean population density (nematodes per 100 cm^3^ of soil) of needle nematode (A) and stunt nematode (B) in irrigated and non-irrigated soils in Michigan corn grown in 2018. Letters on the top of each bar represent significant differences among the soil types (Tukey HSD, α = 0.05).

*Plant-parasitic nematode abundance by irrigation in Michigan corn:* Within this survey, 12% of feed corn growers irrigated their corn. Lower needle nematodes were found in irrigated fields in comparison to non-irrigated soils (*P* > 0.100) (Fig. 7A). However, stunt nematodes were higher in non-irrigated soils (Fig. 7B). Interestingly, lesion nematodes showed no trend related to irrigation.

*Plant-parasitic nematode abundance by cropping system in Michigan corn:* The most common rotation in Michigan is a corn-soybean rotation. During the year of the survey (2018), 63% of surveyed farms had soybeans the year prior. Other crops preceding corn in our study fields were corn, dry beans, kidney beans, pickles, seed corn, winter wheat, and wheat to rye. The average number of lesion, spiral, and stunt nematodes were highest in continuous corn, corn-soybean, and corn-corn rotations. Stunt nematodes were most frequently encountered in systems that had either corn or soybeans in 2017. Across the board, the lowest numbers of PPNs were recovered from corn fields that had dry beans in 2017, followed by fields that included pickles in their rotation. Needle nematodes were recovered from fields with a history of corn, soybean, wheat, and kidney beans the prior year. The number of needle nematodes was also not affected by corn type (field corn or seed corn) (*P* > 0.100). The spiral nematode was the most abundant PPN in all cropping systems.

## Discussion

In this study, ten different genera of PPNs were identified in Michigan corn fields, including lesion, spiral, needle, stunt, dagger, ring, lance, cyst, pin, and root-knot nematodes.

PPNs like lesion, spiral, stunt, dagger, ring, cyst, pin, and root-knot nematode were below thresholds for Michigan corn. However, the corn needle nematode is very destructive and has a threshold of one nematode per field (Niblack, 2003). We found corn needle nematodes in all five regions of Michigan: East/Central, North, Northwest, Southeast, and Southwest (Fig. 3). Berrien, St. Joseph, Alpena, and Presque Isle counties contained more needle nematodes in comparison to other counties, with the highest needle nematode populations extracted from St. Joseph County (11/100 cm^3^). Overall, we uncovered that 26.74% of Michigan corn fields possessed ≥ 1 needle nematode per field in early summer, which is above the threshold. This indicates that the corn needle nematode poses a significant threat to Michigan corn production.

The identities of nematode genera recovered throughout this Michigan corn survey can be compared with results reported across the Midwest. These same genera were recovered in surveys of corn in Iowa and Ohio (Tylka, 2011; Simon, 2018). However, Simon (2018) did not report needle nematodes in Ohio. In a survey of several counties in Illinois, spiral and lesion nematodes were the most abundant within surveyed areas (Han et al., 2021); spiral nematodes were similarly prevalent in our survey of Michigan corn fields.

Plant-parasitic nematode species and population densities are known to be affected by soil type and tillage practices. In this survey, we found that different corn nematodes like lesion, spiral, needle, dagger, and ring nematodes may have different soil preferences. Many nematode surveys have recovered the lowest densities of stunt nematodes from fields with clay soils (Smiley et al., 2004; Chowdhury et al., 2020). In this survey, significantly higher densities of stunt nematodes were found in clay and clay loamy soils, and the lowest numbers were found in sandy and sandy loam soils. Soil moisture has been associated with the presence and abundance of many corn nematodes like needle nematodes and lesion nematodes (Warner, 2011; Smiley, 2021). We found numbers of stunt nematodes were highest in irrigated fields (*P* > 0.100) and needle nematodes were lowest in irrigated fields (*P* < 0.100). Likewise, tillage had mixed results on lesion nematode populations in various agricultural systems: decreased (Govaerts et al., 2007), increased (Thompson, 1992), and not affected (Okada and Harada, 2007). Lesion nematode populations decline with reduced tillage or no-till, in comparison with conventional tillage (Fortunm and Karlen, 1985). In our survey, we also did not observe a clear effect of tillage on lesion nematode abundance; the lowest lesion numbers were encountered in fields with conservation tillage. Surprisingly, no-till for different periods and conventional tillage led to a similar number of lesion nematodes (Fig. 4A). The most needle nematodes were found in corn fields with no-till (Fig. 4C), and the least were found in conservation tillage. Root-knot nematodes were found in only a few counties within our survey; however, it has been reported that tillage in the spring and fall reduces root-knot numbers in corn, compared to a no-till or ridge-till system (Thomas, 1978; McSorley and Gallaher, 1993). It is also notable to mention that sedentary endoparasitic nematodes may be underrepresented within our survey, because root samples were not collected.

Furthermore, PPN abundance can be greatly impacted by the soil type and the sampling date (Norton and Hoffmann, 1975; Tylka et al., 2011). For instance, needle nematodes are often localized and restricted to soils containing at least 49% sand (Norton and Hoffmann, 1975). In this study, needle nematodes were found in clay loam, loam, and sandy loam soil, with the highest abundance detected in sandy loam soil. In comparison to surveys in Illinois, Iowa, and Ohio, more needle nematodes were detected in Michigan corn fields. One possible reason could be the timing of soil samples. Our initial samples were collected in June and harvest samples were collected in October, rather than in June-July like in the Ohio study (Simon, 2018). As discussed previously, needle nematodes will typically move downward in the soil profile during periods of high temperatures, suggesting in June and July they may have been outside the sampling profile for the Ohio survey. Therefore, when sampling for PPNs like needle nematodes, samples should be collected in the spring and early fall (Tylka et al., 2011). Though we note potential trends exist between soil type and tillage on average, none of the factors (i.e., tillage, corn type, irrigation, soil type, corn type, or field history) significantly impacted needle nematode prevalence (*P* > 0.100).

There were a few limitations of our soil survey for plant-parasitic nematode populations in Michigan corn fields. We did not collect root samples, and some PPNs we encountered (like sedentary endoparasites) may not have been feeding on corn. Another potential limitation of this survey could be the method of extraction (sugar centrifugal floatation) or the conditions of the field during collection. However, our protocol that surveyed 86 Michigan corn fields from 20 counties should offer evidence as to which nematodes pose a threat to the northeast portion of the United States corn belt. Soil surveys can help determine potential trends between plant-parasitic nematode abundance and important cropping factors within Michigan corn fields, leading to further research investigating these relationships. Another limitation of our survey is that we did not identify plant-parasitic nematodes to species. Cyst nematodes, for example, may be several species either relevant to corn production (corn cyst nematode), or not relevant (soybean cyst nematode).

From this survey of Michigan corn fields, we conclude that Michigan has a similar profile of plant-parasitic nematode genera to other Midwest corn-producing states (Ohio, Iowa, Illinois): lesion, spiral, needle, stunt, dagger, ring, lance, cyst, pin, and root-knot nematodes. Like the most recent survey conducted in Ohio, we found spiral nematodes to be the most prevalent and abundant plant-parasitic nematode detected in Michigan corn fields (Simon et al., 2018). Spiral nematodes are less pathogenic to corn than needle nematodes, being that it only takes one needle nematode per field to warrant concern. Furthermore, the most significant finding of our survey is that corn needle nematodes were recovered at above threshold populations from all five of the Michigan regions we surveyed, and overall were prevalent in 26% of surveyed corn fields in early summer. Stunt, spiral, and lesion nematodes have higher thresholds of 500-1000 per 100 cm^3^ of soil, yet in certain fields can surpass these levels (MacGuidwin, 2003; Tylka, 2011). Overall, many of the nematodes found in corn are economically important to Michigan’s agriculture systems. However, regarding corn production, we found these nematodes were typically below the listed damage thresholds in our 2018 survey. Some existing management practices that help to keep PPNs lower in numbers are crop rotation with a non-host crop, leaving fields fallow, incorporating cover cropping systems, reducing-tillage practices, and/or applying appropriate nematicides when necessary and economical.
